# Reliability and validity of the short quality of life scale among bank employees in Guangxi, China

**DOI:** 10.3389/fpsyg.2025.1497827

**Published:** 2025-05-21

**Authors:** Qianwei Huang, Qiqing Mo, Huiming He, Xinyu Bai, Jie Zhang, Zhenyu Ma, Guoxiang Chen

**Affiliations:** ^1^School of Public Health, Guangxi Medical University, Nanning, Guangxi, China; ^2^Guilin People’s Hospital, Guilin, Guangxi, China; ^3^Department of Child, Adolescent Health and Maternal Care, School of Public Health, Capital Medical University, Beijing, China; ^4^Guangxi Academy of Medical Sciences, The People's Hospital of Guangxi Zhuang Autonomous Region, Nanning, Guangxi, China; ^5^School of Public Health, Shandong University, Jinan, Shandong, China; ^6^Department of Sociology, State University of New York (SUNY) Buffalo State University, Buffalo, NY, United States; ^7^Life Sciences Institute, Guangxi Medical University, Nanning, Guangxi, China

**Keywords:** quality of life, reliability, validity, suicidal ideation, China

## Abstract

**Background:**

This study aims to assess the reliability and validity of the Quality of Life Scale (QOLS-6) among bank employees in Guangxi, grounding the investigation in the theoretical framework of quality of life measurement and psychological well-being. Given the increasing importance of mental health in the workplace, understanding how psychological conditions impact life quality is critical. The QOLS-6, a widely used tool for measuring quality of life, has been shown to have potential for application in diverse populations.

**Methods:**

A cluster sampling method was used in the study. A questionnaire survey was conducted among 3,974 employees of a bank in Guangxi Province of China. To evaluate its performance across different mental health conditions, 298 participants in the suicidal ideation group and 3,676 in the non-suicidal ideation group. The non-suicidal group was randomly divided into two subsamples, with subsample A (*n* = 1,838) for exploratory factor analysis (EFA) and subsample B (*n* = 1,838) for confirmatory factor analysis (CFA). Reliability and validity were assessed for each group, enhancing the understanding of QOLS-6’s sensitivity and effectiveness in capturing quality of life variations across different psychological profiles. Cronbach’s *α* coefficient was used to analyze the internal consistency reliability. EFA and CFA were used to examine the construct validity. Spearman correlations was used to evaluate the concurrent validity.

**Results:**

The QOLS-6 demonstrated good internal consistency, with Cronbach’s *α* values of 0.908 and 0.857 for the two groups, respectively, and 0.865 for the overall sample. Its construct validity was supported by high KMO values of 0.841, 0.805 and 0.818 for the suicidal ideation group, subsample A and total sample, respectively. Exploratory factor analysis of the QOLS-6 revealed a single-factor structure. However, confirmatory factor analysis demonstrated that a three-factor model provided a better fit. The QOLS-6 showed negative correlations with depression, loneliness, hopelessness, and job burnout while positively correlating with job satisfaction and family function.

**Conclusion:**

The QOLS-6 has demonstrated strong reliability and validity among bank employees in Guangxi, China. While exploratory factor analysis suggested a one-factor structure, confirmatory factor analysis supported a three-factor model, indicating a multidimensional nature. Based on the theoretical framework of quality of life and the design of the scale’s content, the three-factor model demonstrates statistical and theoretical validity. Additionally, the scale exhibited significant correlations with key psychological factors, further supporting its applicability. These findings suggest that the QOLS-6 is an effective tool for assessing quality of life in diverse psychological contexts.

## Introduction

The World Health Organization defines quality of life as an individual’s subjective evaluation of their position in the cultural and value system, as well as their relationships with their goals, expectations, standards, and concerns (WHOQOL, 1993). Within various demographic groups and across diverse cultural milieus, the quality of life plays a significant role in shaping suicidal ideation and behaviors have been reported by researches (He et al., 2020). Health-related quality of life is affected by psychological issues such as depression and suicidal ideation which has been shown significantly in stroke patients in the United States (Chen et al., 2024). In Nigeria, suicidal behavior among HIV-infected individuals is strongly associated with a diminished quality of life (Oladeji et al., 2017). Similarly, a clear link between decreased quality of life and suicidal ideation have been identified by the study in a Australian general community (Goldney et al., 2001). These findings underscore the close relationship between quality of life and suicidal ideation. Therefore, this study groups participants based on the presence or absence of suicidal ideation to more accurately assess quality of life across different psychological states. Assessing quality of life provides a comprehensive understanding of an individual’s physical, psychological, and social well-being, which are closely linked to suicidal ideation (Ki et al., 2024). The quality of life scale serves as an effective tool for identifying individuals experiencing a diminished quality of life, who may be at risk for mental health issues and potentially suicidal ideation or behaviors (Chen et al., 2024). This allows for early detection and targeted intervention to address underlying issues such as depression, loneliness, and hopelessness, thereby reducing the likelihood of suicidal behavior.

The Guangxi region in China, situated in the multi-ethnic and less developed western part of the country. In recent years, banking professionals have been experiencing significant psychological stress because of the pressures associated with digital transformation and other factors (Li, 2022). A study reported that the prevalence of suicidal ideation among bank employees in Guangxi was 7.5%, with major depression, hopelessness, and negative coping styles identified as significant risk factors (He et al., 2020).

The quality of life of Chinese bank employees is influenced by multiple factors and exhibits significant regional disparities. Work-life imbalance is a critical factor contributing to the decline in their quality of life. Long hours in confined environments and strict performance evaluations, which are typical of the banking profession, severely disrupt the work-life balance of bank employees. Research has demonstrated a significant positive correlation between work-life balance and job satisfaction in the Shanghai region (Xue et al., 2024). Due to factors such as fewer bank branches, employees in county and rural areas are more susceptible to work-life imbalance, leading to a decline in their quality of life (Li and Liu, 2021). Environmental factors, particularly the work environment, have a substantial impact on the quality of life of bank employees (Gür et al., 2016). Furthermore, grassroots bank employees in different altitude regions show varying probabilities of abnormal results in physical examinations, which further affect their quality of life (Wang and Zhao, 2008). These disparities suggest that improving the quality of life of Chinese bank employees requires tailored strategies and a comprehensive consideration of various factors. Nonetheless, research on the quality of life of bank employees remains limited, especially regarding the validation of relevant assessment tools.

Various measurement tools provide quantitative bases for assessing the quality of life of bank employees. WRQoL scale focuses on work-related aspects of life quality, accurately capturing employees’ quality of life within their work environment (Geçkil, 2021). The Quality of Work Life Scale measures multiple dimensions, including job satisfaction, overall well-being, and work stress (Tentama and Suwandi, 2020). The scale was evaluated using structural equation modeling, which demonstrated that its dimensions and indicators effectively captured the construction of work life quality with high validity. In the empirical analysis of bank employees’ quality of life, Cronbach’s *α* was employed to assess the reliability of the model, and the results indicated a satisfactory level of reliability. Although various quality of life scales, including QOLS (Burckhardt and Anderson, 2003), WHOQOL-BREF (Makovski et al., 2019), CASP-11-SG (Tan et al., 2023), CQ-11D (Zhou et al., 2024) and ProQOL (Galiana et al., 2020), each has certain limitations. The WHOQOL-BREF demonstrates strong cross-cultural validity but requires lengthy administration time, while the QOLS, CASP-11-SG and CQ-11D face challenges in cultural adaptation within Chinese contexts. ProQOL, while effective in assessing professional burnout, has a narrower focus. Given these limitations, we selected the QOLS-6 scale and conducted targeted validation to better suit the specific population of this study.

This study used the 6-item short form Quality of Life Scale (QOLS-6) developed by Phillips (Phillips et al., 2002). The QOLS-6 was showed good reliability and validity in a psychological analysis of the general Chinese population in 2002. The scale focuses on six dimensions of quality of life over the preceding 30 days, including physical well-being, psychological health, economic status, occupational satisfaction, family relationships, and social interactions. The QOLS-6 has been validated in a study of psychological autopsy of elderly individuals who died by suicide in rural China, demonstrating its reliability and effectiveness within this population (He et al., 2021). Currently, the QOLS-6 has demonstrated strong psychometric properties primarily among rural elderly populations in China, highlighting its potential for broader applicability. The scale features a concise structure with only six items, enhancing its practicality for large-scale assessments.

To the best of our knowledge, QOLS-6 was used for the first time in research among the bank employees. This study evaluates the applicability of the QOLS-6 within bank employees in Guangxi, providing a foundation for future research to validate its generalizability across diverse occupational and regional groups. By demonstrating its reliability and validity, the study proposes the QOLS-6 as a practical tool for assessing quality of life in mental health research contexts. The following research hypotheses are proposed:

*H1*: Based on the theoretical framework of quality of life and the design of the scale’s content, three latent factors can be extracted from the quality of life scale in the bank employee population.

*H2*: The quality of life scale demonstrates good reliability in the bank employee population.

*H3*: The quality of life scale demonstrates good validity in the bank employee population.

## Methods

### Design and participants

In this study, a cross-sectional survey and cluster sampling method were adopted to conduct an online questionnaire survey among 5,000 employees of a bank in Guangxi from November to December 2019. Guangxi is an underdeveloped region in western China. The research sample covers several prefecture-level cities such as Nanning, Liuzhou, Guilin and Guigang. The work institutions involved include first and second-level branches and first and second-level sub-branches of banks, and the work institution levels involved cover both the front desk and middle and back offices of banks. The researchers sent each respondent a survey link via an electronic questionnaire and briefly introduced the study. The participants signed informed consent, and their identities remained anonymous prior to the commencement of the study. To prevent data loss and redundant responses, it was necessary for each respondent to complete all questions and submitted their response only once. Meanwhile, the survey included an automated logic verification feature to guarantee the integrity of responses. After the survey was completed, the data was double-checked, cleaned, and reviewed, resulting in the collection of 3,974 valid questionnaires, including 298 employees in the suicidal ideation group and 3,676 employees in the non-suicidal ideation group. This study was approved by the Medical Ethics Committee of Guangxi Medical University (Ethical Application Ref: KY20240151).

### Measurements

#### Demographics

The collected demographic data includes sex, age, educational level (Associate Degree or below, Bachelor’s Degree, Master’s Degree or above), marital status (Single, Married, or Other), and years of working experience (≤3 years, 4–12 years, over 12 years). “Other” marital statuses include separation, divorce, and widowhood. The age grouping is based on the sample’s median age of 35 years. Work experience groups are defined according to the requirement in China for new employees to sign a labor contract of at least three years at the beginning of their employment, combined with the division of career stages into early, mid, and late phases.

#### Quality of life scale (QOLS-6)

The QOLS-6 developed by Phillips (Phillips et al., 2002) assesses the respondent’s quality of life in six dimensions over the preceding month: physical, psychological, economic, occupational, familial relationships, and social interactions. Participants rated each item on a 5-point Likert scale (1 = very poor, 5 = excellent), with total scores ranging from 6 to 30. A higher total score indicates a better quality of life.

#### Suicidal ideation and self-harming actions

The presence of suicidal ideation within the past year is assessed by asking the question “Have you seriously considered ending your own life within the past year?” (Wang, 2019). In addition, self-harming actions were evaluated with the question: “At any point in the past, have you taken any self-harming actions?” Participants responded with either “Yes” or “No.”

#### Maslach burnout inventory-general survey (MBI-GS)

The scale measures three dimensions of emotional exhaustion, depersonalization, and diminished professional accomplishment. This is a self-report questionnaire with 15 items, rating on a 7-point scale (0 = never to 6 = each day). The higher the total score on the scale, the higher the level of job burnout (Li and Shi, 2003). The scale demonstrated good internal consistency, with a Cronbach’s *α* coefficient of 0.884.

#### Job satisfaction scale

The scale consists of six items, including satisfaction with promotion opportunities, colleagues, superiors, the job itself, income, and overall satisfaction. The answers can be given using a 5-point Likert Scale (1 = very dissatisfied to 5 = very satisfied). The higher the total score of the scale, the more satisfied the job (Schriesheim and Tsui, 1980). The Cronbach’s *α* for the scale was 0.865, indicating satisfactory reliability.

#### Patient health questionnaire (PHQ-9)

PHQ-9 consists of nine depressive symptoms items that evaluate the state of the past two weeks, including both emotional (Items 1, 2, 4, 6, 8, 9) and physical (Items 3, 5, 7) dimensions. The specific items include “Over the past two weeks, how often have you felt little interest or pleasure in doing things?,” etc. The answers can be given using a 4-point Likert Scale ranging from none to every day. The total score of the scale is 27 points, The higher total score indicating more severe depressive symptoms (Spitzer et al., 1999). In the study, the Cronbach’s *α* for the scale was 0.918.

#### Chinese revision of short-form of the UCLA loneliness scale (ULSA-6)

Consisting of 6 items, it uses a 4-point Likert Scale (1 = never, 5 = always). The specific items include “Lack of company from others,” “No one to turn to for help,” “I feel neglected,” etc. The total score of the scale ranges from 6 to 24 points, with higher scores indicating a stronger sense of loneliness (Zhou et al., 2012). The Cronbach’s *α* for the scale was 0.913.

#### Beck hopeless scale (BHS)

The BHS evaluates an individual’s mental state over the previous week, comprising four items rated on a 5-point Likert Scale from completely agree to completely disagreement. The specific items include, “I hope that in the future I can do the most important things well.,” “My future is dark.,” “I’m just unlucky and I’ll always be.” and “I am full of confidence about the future.” The total score ranges from 4 to 20, with higher scores indicating a greater sense of hopelessness (Beck et al., 1974). The Cronbach’s *α* for the scale was 0.797.

#### Affection and resolve scale (family APGAR)

The study utilized the Family APGAR Questionnaire developed by Smilkstein (1978), which evaluates family functionality across five dimensions: Adaptation, Partnership, Growth, Affection, and Resolve. The questionnaire employs a 3-point reverse scoring system, with responses of “often,” “sometimes,” and “rarely” scored as 2, 1, and 0 points, respectively. The total score ranges from 0 to 10, with higher scores indicating better family functionality. The Cronbach’s *α* for the scale was 0.874.

### Statistical analysis

Categorical variables were expressed as count and percentage, while continuous variables were expressed as mean ± standard deviation (Means ± SD). Descriptive analysis, chi-square test, independent sample t-test and Mann–Whitney test were employed to compare demographic characteristics between the groups.

Reliability was assessed using Cronbach’s α coefficient, where values between 0.70 and 0.79 were deemed acceptable, 0.80 to 0.89 as good, and ≥ 0.90 as excellent (van Krugten et al., 2022). Corrected item-total score correlations were also calculated, with coefficients≥0.40 considered indicative of acceptable item consistency within the overall scale.

To ensure the robustness of the factor structure, the non-suicidal ideation group (*N* = 3,676) was randomly split into two subsamples: Subsample A (*n* = 1,838) for EFA and Subsample B (*n* = 1,838) for CFA. The structural validity of QOLS-6 was assessed through exploratory factor analysis (EFA). The suitability of EFA was confirmed by the Kaiser-Meyer-Olkin (KMO) measure of sampling adequacy and Bartlett’s test of sphericity, where KMO values >0.600 and *p* < 0.05 indicated adequacy (Ma et al., 2020). EFA was conducted using principal components extraction and varimax rotation, with factors identified based on eigenvalues >1 and the scree plot. A factor loading cut-off of ≥0.40 was applied to retain items contributing significantly to the factor structure. To ensure the accuracy of the results, a parallel analysis using Monte Carlo simulation was conducted, generating a series of data sets that simulate the experimental data. If the eigenvalues of the factors from the experimental data set are larger than those for the simulated data set, then it can be concluded that the respective factors are present in the data set (Sanatkar and Rubin, 2023). Confirmatory factor analysis (CFA) was used to evaluate the construct validity. Using chi-square test, root mean square error of approximation (RMSEA; values<0.08 are acceptable, ≤0.05 are ideal), standardized root mean square residual (SRMR; values ≤0.05 suggest a good fit), Bentler Comparative Fit Index (CFI), and Bentler Bonett normed fit index (NFI) (CFI and NFI values >0.90 suggest adequate fit) (Sun et al., 2019). For concurrent validity, Spearman correlation was used, with correlation coefficients below 0.2 considered absent, 0.2 to <0.35 as weak, 0.35 to <0.50 as moderate, and ≥ 0.50 as very strong (Haspels et al., 2023). Statistical analyses were performed using SPSS 27.0, Amos 26.0 and Mplus 8.3 software, and a two-tailed *p*-value of <0.05 was considered statistically significant.

## Results

### Descriptive analysis

#### Demographics

Among the 3,974 survey participants, there were 1,195 males (30.1%) and 2,779 females (69.9%). Among them, 298 (7.5%) reported suicidal ideation, as a suicidal ideation group, 3,676 (92.5%) of non-suicidal ideation workers as a non-suicidal ideation group. As shown in Table 1, it is evident that the prevalence of suicidal ideation is significantly higher among individuals aged ≤35 years (*p* = 0.001), and with 4–12 years of work experience (*p* = 0.038). The prevalence of self-harming actions was found to be higher among participants with suicidal ideation (*p* < 0.001). The scores of depression, hopelessness, loneliness, and job burnout in the group with suicidal ideation were significantly higher than those in the non-suicidal ideation group (*p* < 0.001), while their scores of job satisfaction and family function were significantly lower than those in the non-suicidal ideation group (*p* < 0.001).

**Table 1:** Comparison of different characteristics between the suicidal ideation group and non-suicidal ideation group.

Characteristics	Suicidal Ideation Group (*N* = 298)	Non-suicidal Ideation Group (*N* = 3,676)	χ^2^*/Z/t*	*p*
Sex			0.704	0.401
Male	96 (32.2)	1,099 (29.9)		
Female	202 (67.8)	2,577 (70.1)		
Age groups			−3.783	**0.001**
≤35	194 (65.1)	1976 (53.8)		
>35	104 (34.9)	1700 (46.2)		
Years of working			−2.079	**0.038**
≤3 years	22 (7.4)	357 (9.7)		
4–12 years	201 (67.4)	2072 (56.4)		
>12 years	75 (25.2)	1,247 (33.9)		
Educational level			0.218	0.897
Associate degree or below	70 (23.5)	845 (23.0)		
Bachelor’s degree	223 (7.4)	2,780 (92.6)		
Master’s degree or above	5 (8.9)	51 (91.1)		
Marital status			5.855	0.054
Single	56 (18.8)	529 (14.4)		
Married	222 (74.5)	2,953 (80.3)		
Other	20 (6.7)	194 (5.3)		
Self-harming actions			−11.577	**<0.001**
Yes	99 (33.2)	55 (1.5)		
No	199 (66.8)	3,621 (98.5)		
Depression (Means ± SD)	11.51 ± 7.88	7.25 ± 5.19	9.180	**<0.001**
Hopeless (Means ± SD)	10.57 ± 3.40	8.26 ± 2.93	12.955	**<0.001**
Loneliness (Means ± SD)	14.86 ± 5.13	12.27 ± 4.33	8.464	**<0.001**
Job Burnout (Means ± SD)	42.99 ± 13.92	35.53 ± 12.22	8.982	**<0.001**
Job satisfaction (Means ± SD)	19.15 ± 3.95	20.66 ± 3.31	−6.391	**<0.001**
Family APGAR (Means ± SD)	5.68 ± 3.11	7.07 ± 2.65	−7.496	**<0.001**

APGAR, family adaptive, partnership, growth, affection and resolve scale. Bold values indicate *p* < 0.05.

#### Score of each item in quality of life scale

As shown in Table 2, total scores of quality of life in the suicide ideation group and the non-suicide ideation group were 18.59 ± 5.23 and 20.44 ± 3.80, respectively. Compared with the non-suicidal ideation group, the suicide ideation group had lower quality of life scores, indicating that the quality of life of the suicide ideation group was worse than that of the non-suicidal ideation group (*p* < 0.001).

**Table 2:** The score of each item for suicidal ideation group and non-suicidal ideation group in QOLS-6.

Item	Suicidal Ideation Group (Means ± SD)	Non-suicidal Ideation Group (Means ± SD)	*t*	*p*
1. How was your physical health in the last month?	2.92 ± 1.13	3.22 ± 0.95	−4.453	<0.001
2. How was your psychological health in the last month?	2.80 ± 1.19	3.30 ± 0.93	−7.135	<0.001
3. How was your economic status in the last month?	2.81 ± 1.08	2.98 ± 0.81	−2.659	0.008
4. How was your work in the last month?	3.07 ± 1.00	3.32 ± 0.78	−4.149	<0.001
5. How were your relationships with your family in the last month?	3.50 ± 1.00	3.87 ± 0.80	−6.194	<0.001
6. How were your relationships with others in the last month?	3.49 ± 0.91	3.76 ± 0.70	−4.992	<0.001
Total scores	18.59 ± 5.23	20.44 ± 3.80	−5.986	<0.001

### Reliability

#### Internal consistency reliability

The Cronbach’s *α* coefficient of the QOLS-6 scale was 0.865. For the suicidal ideation group and the non-suicidal ideation group, the Cronbach’s α coefficients were 0.908 and 0.857, respectively. This indicates good internal consistency reliability.

#### Corrected item-total score correlation

The correlation coefficients between each item of QOLS-6 and the total scores of the suicidal ideation group and the non-suicidal ideation group were 0.673–0.829 and 0.512–0.769, respectively. Cronbach’s α coefficients if item deleted ranged from 0.879–0.902 among suicidal ideation group and varied from 0.807–0.856 among non-suicidal ideation group. The details were shown in Table 3.

**Table 3:** Corrected part-whole correlations and Cronbach’s α if item deleted of QOLS-6 among suicidal ideation group and non-suicidal ideation group.

Items	Total Sample		Suicidal Ideation Group		Non-suicidal Ideation Group	
	*R*	*α*	*R*	*α*	*R*	*α*
1. How was your physical health in the last month?	0.678	0.840	0.751	0.891	0.666	0.830
2. How was your psychological health in the last month?	0.780	0.819	0.829	0.879	0.769	0.807
3. How was your economic status in the last month?	0.536	0.864	0.689	0.9	0.512	0.856
4. How was your work in the last month?	0.705	0.835	0.800	0.884	0.689	0.825
5. How were your relationships with your family in the last month?	0.612	0.851	0.673	0.902	0.598	0.841
6. How were your relationships with others in the last month?	0.678	0.842	0.751	0.892	0.663	0.832

### Validity

#### Construct validity

##### Exploratory factor analysis

For the overall sample, the KMO value was 0.818, and Bartlett’s test of sphericity: χ^2^ = 12160.210, *p* < 0.001. The exploratory factor analysis was determined to be suitable for both the suicidal ideation group (KMO = 0.841, Bartlett’s test of sphericity: χ^2^ = 1264.004, *p* < 0.001) and the subsample A (KMO = 0.805, Bartlett’s test of sphericity: χ^2^ = 5208.881, *p* < 0.001). These results indicate that the data met the necessary criteria for factor analysis, with adequate sampling adequacy and significant correlations among variables.

**Figure 1:**
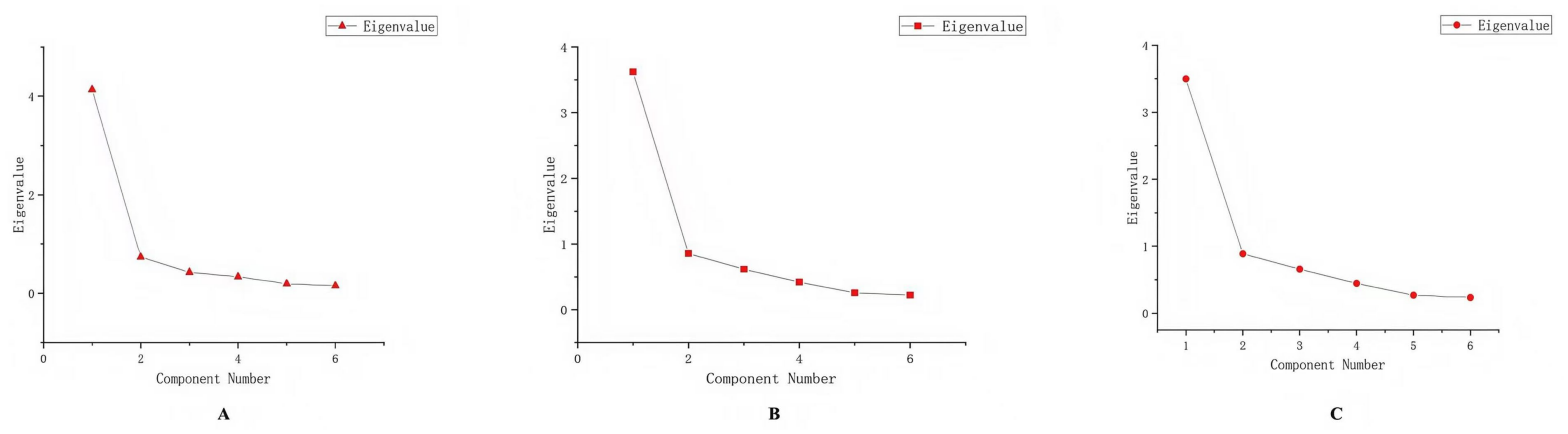
Scree plots from the exploratory factor analysis for **(A)** the total sample, **(B)** the suicidal ideation group, and **(C)** subsample A.

**Figure 2:**
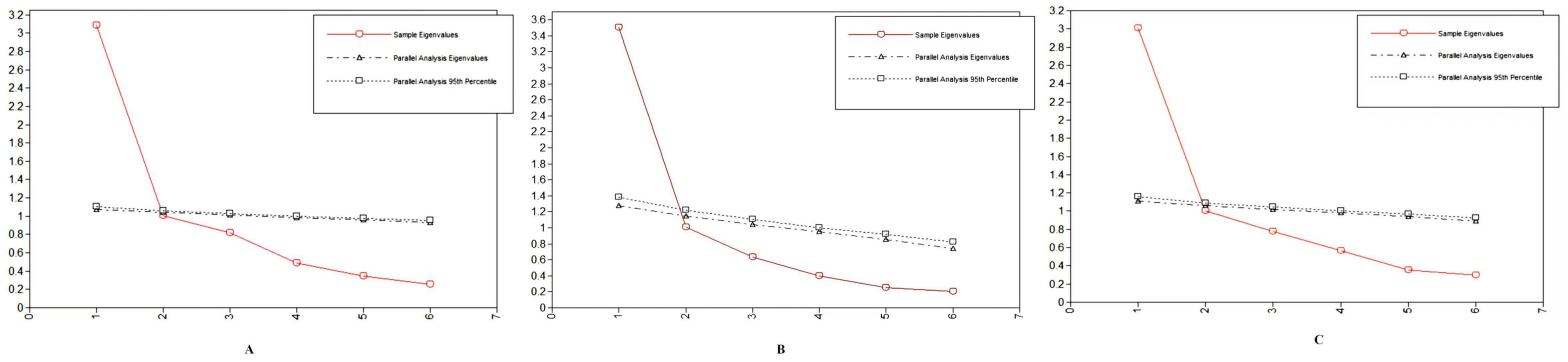
Parallel analysis plots from the exploratory factor analysis for **(A)** the total sample, **(B)** the suicidal ideation group, and **(C)** subsample A.

One common factor was extracted using exploratory factor analysis. The cumulative variance contribution rate was 60.37% for the total sample, 69.00% for the suicide ideation group, and 58.32% for the subsample A, respectively. Exploratory factor analysis extracted one common factor, with Eigenvalue >1.0 and a clear inflection point in the scree plot. Scree plots of exploratory factor analysis of total sample, suicidal ideation group and subsample A are shown in Figures 1A–C. The results of the parallel analysis showed that the eigenvalue of the first factor exceeded the eigenvalue of the first factor in the simulated data set. However, the eigenvalue of the second factor did not exceed the second factor in the simulated data. Consequently, we specified the extraction of only one factor using the promax method of oblique rotation. As shown in Figures 2A–C. Component matrix for each item of the QOLS-6 is shown in Table 4.

**Table 4:** Component matrix for each item of the QOLS-6.

Items	Total sample	Suicidal ideation group	Non-suicidal ideation group
	1	1	1
1. How was your physical health in the last month?	0.783	0.828	0.777
2. How was your psychological health in the last month?	0.86	0.888	0.854
3. How was your economic status in the last month?	0.659	0.778	0.639
4. How was your work in the last month?	0.807	0.869	0.797
5. How were your relationships with your family in the last month?	0.747	0.780	0.739
6. How were your relationships with others in the last month?	0.791	0.835	0.783

##### Confirmatory factor analysis

**Figure 3:**
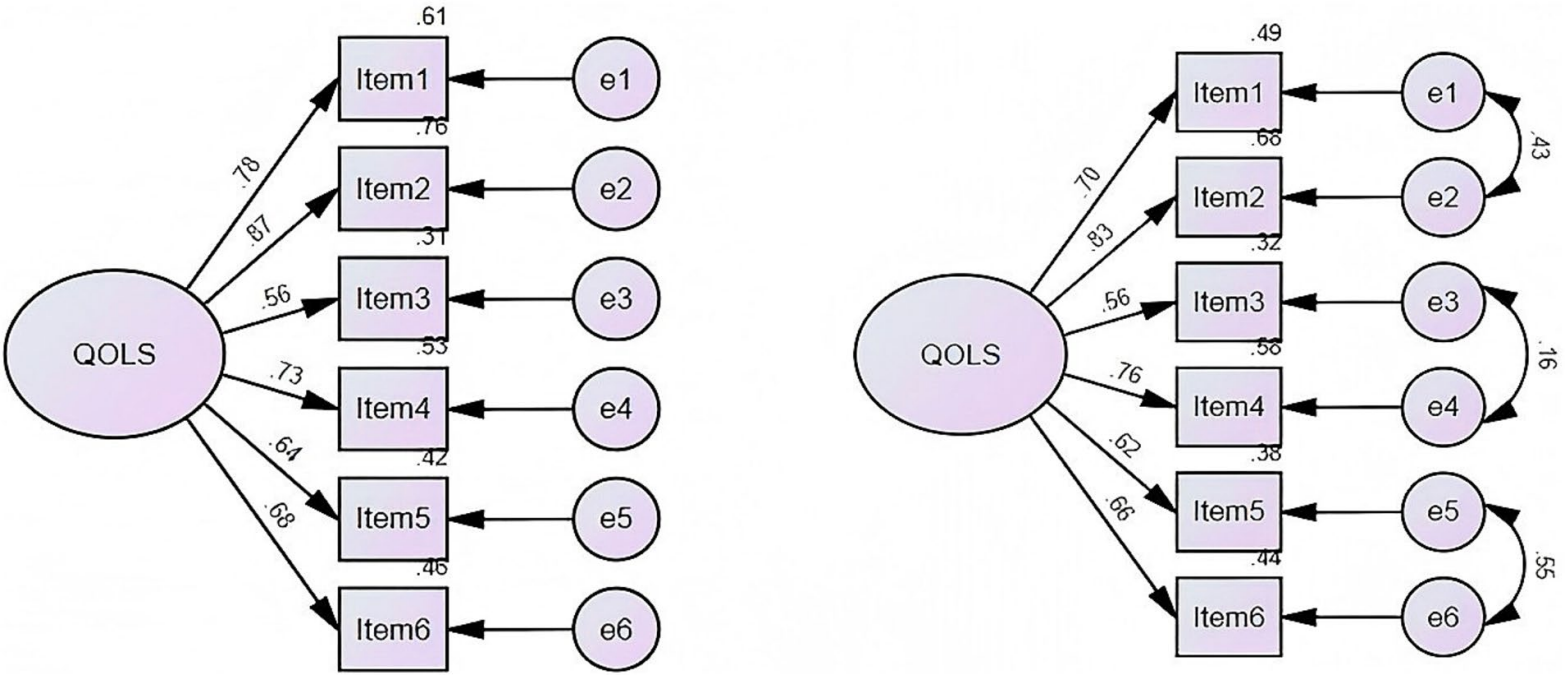
Pathway diagram of the confirmatory factor analysis of the one-factor (left panel) and three-factor (right panel) model for the QOLS-6.

CFA was conducted on subsample B (*n* = 1838). As shown in the left panel of Figure 3, the fit indices for the single-factor model did not meet satisfactory standards (χ^2^ = 891.837, *p* < 0.001, CFI = 0.838 NFI = 0.837, SRMR = 0.048, RMSEA = 0.231). Considering that quality of life generally encompasses physical and mental health, economic status, and interpersonal relationships (The WHOQOL Group, 1998), and based on the specific content of the scale, correlations were added between items 1 and 2, items 3 and 4, and items 5 and 6. The fit indices improved (χ^2^ = 33.757, *p* < 0.001, CFI = 0.995, NFI = 0.994, SRMR = 0.012, RMSEA = 0.050). As shown in the right panel of Figure 3. Consequently, confirmatory factor analysis suggested that a three-factor solution was superior to a one-factor structure.

#### Concurrent validity

Table 5 shows significant correlations between QOLS-6 and other scales. The quality of life among bank employees is positively associated with family function and job satisfaction (*p* < 0.001), while showing negative associations with job burnout, depression, loneliness, and hopelessness (*p* < 0.001). In both the total sample and the suicidal ideation group, QOLS-6 exhibits a strong correlation with all other scales (*p* < 0.01). In the non-suicidal ideation group, QOLS-6 is strongly positively correlated with MBI-GS, PHQ-9, ULAS-6, and BHS-4 (*p* < 0.01), and moderately negatively correlated with APGAR and Job Satisfaction (*p* < 0.01).

**Table 5:** Correlation coefficient between the QOLS-6 and other relevant scales.

Scales	QOLS-6		
	Total sample	Suicidal ideation group	Non-suicidal ideation group
	*r*	*r*	*r*
MBI-GS	−0.533^***^	−0.600^***^	−0.513^***^
PHQ-9	−0.633^***^	−0.732^***^	−0.601^***^
ULAS-6	−0.571^***^	−0.689^***^	−0.538^***^
BHS-4	−0.513^***^	−0.503^***^	−0.506^***^
APGAR	0.513^***^	0.516^***^	0.496^***^
Job Satisfaction	0.503^***^	0.586^***^	0.466^***^

QOLS-6, Quality of life scale; MBI-GS, Maslach burnout inventory-general survey; PHQ-9, Patient health questionnaire; ULAS-6, University of California Los Angeles Loneliness Scale-6; BHS-4, Beck’s hopelessness scale; APGAR, family adaptive, partnership, growth, affection and resolve scale. ***p < 0.001.

## Discussion

The results of this study showed that QOLS-6 had good reliability and validity. QOLS-6 is a practical tool to assess the quality of life among the bank occupational population.

The scale was also utilized in a psychological autopsy study conducted among elderly individuals at risk of suicide in rural China (He et al., 2021), demonstrating commendable reliability and validity. However, the mean quality of life scores observed within both the suicide group and non-suicidal ideation group were comparatively lower than those reported in this study, potentially attributable to varying conditions influencing quality of life across diverse groups. Consequently, it is imperative to conduct specific analyses when formulating intervention strategies. The study shows good internal consistency of QOLS-6 in overall sample, and both in the suicidal ideation group and the non-suicidal ideation group. One of the best methods for evaluating internal consistency is Cronbach’s *α* coefficient, which were used to assess the reliability of QOLS-6. The Cronbach’s α coefficient of the QOLS-6 scale was 0.865. For the suicidal ideation group and the non-suicidal ideation group, the Cronbach’s α coefficients were 0.908 and 0.857, respectively. Also, QOLS-6 has good uniformity and stability in this study. The similar result was reported in the research among suicide elderly (He et al., 2021).

The split-sample EFA-CFA design within the non-suicidal group provides rigorous evidence for the unidimensionality of QOLS-6. The exploratory factor analysis supports a single-factor structure for the QOLS-6, with the analysis revealing only one common factor. This result differs from the finding of two factors identified in the research on elderly people who committed suicide in rural areas (He et al., 2021). Such a discrepancy may be attributed to differences in the characteristics of the populations studied. Confirmatory factor analysis revealed a three-factor structure (physical-mental health, economic-occupational status, and family-social relationships), which significantly improved model fit compared to the original single-factor model (CFI = 0.995 vs. 0.838, RMSEA = 0.050 vs. 0.231). This multidimensional structure aligns with the theoretical framework of quality of life proposed by the World Health Organization. Furthermore, the improved fit indices of the adjusted model suggest that incorporating correlations between items allows for a better capture of the underlying relationships between dimensions. A possible reason why EFA failed to extract three factors is the high correlation among measurement items. When different dimensions of the scale are closely related, EFA may underestimate the number of factors, leading to discrepancies between the factor structures identified by EFA and CFA (Sanatkar and Rubin, 2023). Moreover, EFA is a data-driven approach that assumes factors are uncorrelated by default (Mindrila, 2017). However, different dimensions of quality of life are often interrelated. As a result, EFA may fail to identify an underlying multifactor structure. In contrast, CFA is theory-driven and tests a predefined factor structure based on prior theoretical support. By accounting for correlations among factors, CFA provides a more accurate representation of the theoretical framework. Therefore, the findings support the classification of this scale into three distinct factor dimensions. This finding further validates the interrelationships among the different dimensions of the scale and provides valuable insights for future improvements to the scale.

In this study, quality of life was positive with family function and job satisfaction, negative with job burnout, depression, loneliness, and hopelessness. Depression can seriously lower the quality of life (Malhi and Mann, 2018). The sense of loneliness among elderly in China is negatively correlated with their quality of life (Zhu et al., 2018). Despair plays a mediating role between quality of life and suicide (Chen et al., 2022). A high quality of life reduces depression, hopelessness and suicidal ideation (Büsselmann et al., 2019). Good family function can improve the quality of life for older adults (He et al., 2021). Previous studies have shown similar results, so based on the correlation between these factors, the QOLS-6 also demonstrates good concurrent validity.

Quality of life is closely related to suicidal ideation, and a deep understanding of the relationship between them is helpful to prevent suicide. A large number of studies at home and abroad have proved that suicidal ideation is associated with low quality of life, including rural elderly people (He et al., 2021), community residents (van Spijker et al., 2020), Parkinson’s patients (Santos-García et al., 2023) and so on. In this study, it shows that employees with suicidal ideation have a lower quality of life.

This study, by examining the reliability and validity of the QOLS-6 among bank employees in Guangxi, particularly distinguishing between groups with and without suicidal ideation, demonstrates the scale’s applicability and reliability across different psychological states, holding significant practical implications. The findings not only validate the broad applicability of the scale in mental health assessments but also provide an effective tool for monitoring and intervention in high-stress occupational groups, such as bank employees. Through scientific evaluation of employees’ quality of life, management can promptly identify and address potential mental health issues, enabling targeted support and intervention measures to enhance overall quality of life and job satisfaction.

This study also has limitations. Due to the use of a self-administered questionnaire, the study participants and subjectivity may cause certain bias in data quality and accuracy. Secondly, this study focuses on the bank workers in Guangxi, and further investigation is needed for other groups or countries of people, and more other factors should be taken into consideration. The study aimed to verify the reliability and validity of the QOLS-6 among bank employees, and thus did not conduct an in-depth analysis of the population with suicidal ideation. Future research could explore this topic more extensively, which would help to more comprehensively reveal the characteristics and psychological mechanisms of individuals with suicidal ideation, thereby providing a more scientific basis for formulating targeted intervention strategies.

## Conclusion

This study, based on the theoretical framework of quality of life and the design of the scale’s content, measured and analyzed the quality of life of bank employees. Confirmatory factor analysis (CFA) indicated that three latent factors could be extracted from the quality of life scale in this population, and the three-factor model exhibited a better fit than the single-factor model, aligning with theoretical expectations. Additionally, validity testing demonstrated that the scale performed well in terms of concurrent validity. Finally, reliability analysis showed that the internal consistency reliability of the scale reached a high level.

In conclusion, this study confirmed the applicability of the quality of life scale among bank employees in Guangxi, demonstrating its good reliability and validity. The scale can serve as an effective tool for assessing the quality of life of bank employees. However, further assessments of its reliability and validity across different populations and regions are recommended to better evaluate its factor structure.

## Data Availability

The raw data supporting the conclusions of this article will be made available by the authors, without undue reservation.
